# Intraspecific Motor and Emotional Alignment in Dogs and Wolves: The Basic Building Blocks of Dog–Human Affective Connectedness

**DOI:** 10.3390/ani10020241

**Published:** 2020-02-03

**Authors:** Elisabetta Palagi, Giada Cordoni

**Affiliations:** 1 Ethology Unit, Department of Biology, University of Pisa, Via Volta 6, 56126 Pisa, Italy; 2 Natural History Museum, University of Pisa, Via Roma 79, Calci, 56011 Pisa, Italy; giada.cordoni@unipi.it

**Keywords:** motor resonance, facial displays, body signals, synchronization, yawn contagion, relationship quality, play fairness

## Abstract

**Simple Summary:**

It is now widely accepted that animals may express and perceive emotions. This capacity has an adaptive value because it allows animals to respond to various situations quickly and appropriately thus facilitating their survival and increasing their reproductive success. Through spontaneous mimicry, animals can share their emotional mood and this appears to be particularly fruitful when the relationships are not inhibited by rank rules and when animals build and maintain their bonds through cooperation and social affiliation. Dogs represent a very good model to test hypotheses about the importance of mimicry in regulating emotional sharing because they can be tested at both intra- and inter-specific levels. The intra-specific evidence will help us to understand what the social cognitive potential is at the basis of the evolution of the emotional “intimacy” between dogs and their human companions.

**Abstract:**

Involuntary synchronization occurs when individuals perform the same motor action patterns during a very short time lapse. This phenomenon serves an important adaptive value for animals permitting them to socially align with group fellows thus increasing integration and fitness benefits. Rapid mimicry (RM) and yawn contagion (YC) are two behavioral processes intermingled in the animal synchronization domain. Several studies demonstrated that RM and YC are socially modulated being more frequently performed by individuals sharing close relationships. This evidence highlights the relation between RM/YC and emotional contagion that is the capacity of two or more individuals to share the same affective state. In this review, we try to delineate a possible developmental trajectory of emotional sharing phenomena by using, as a model species, the domestic dog (*Canis lupus familiaris*), a valid example of empathic predisposition towards individuals belonging both to the same and the different species. We contrast available findings on RM and YC in dog–dog and dog–human dyads with those in wolf–wolf dyads, in order to investigate if the ability to emotionally engage with conspecifics (wolf–wolf and dog–dog) is evolutionary rooted in canids and if provides the basis for the development of inter-specific emotional sharing (dog–human).

## 1. Introduction

Behavioral non-conscious synchronization is widespread among animals, including humans. It occurs when individuals engage in the same motor action during a very tight time window and when they are spatially close [1,2]. Synchronizing with others has a highly adaptive value and produces many different benefits as a function of the type of synchronization. For example, the synchronization in reproduction, movements, and vigilance of group living animals can increase their effectiveness in the defense from predators [1]. Synchronization can occur not only at group level but also at dyadic level [3]. During their dyadic social interactions, human and non-human animals can synchronize their motor actions such as facial expressions and body postures [4]. For example, it has been recently demonstrated that dogs synchronized their resting and locomotor activity with that of their owners when they were freely acting in an open area [2].

The behavioral synchronization domain includes two phenomena that are still debated topics in human and non-human animal studies: Rapid Mimicry (RM) and Yawn Contagion (YC) [5]. Even though they are often considered as different processes, they seem to share similar neural and social mechanisms [6,7,8]. It has been proposed that these motor resonance behaviors are grounded in the automatic perception–action coupling in the sensorimotor areas [5,9] The mirror neurons, discovered in monkeys and humans, provided neurophysiological support to the perception–action model, because they fire when the motor action is both observed and perceived. For this reason, a large array of studies indicates that RM and YC may be automatic, fast, reflex-like mechanisms which go beyond the intentional control [10]. 

RM and YC seem to intermingle with emotional contagion which is defined as “the tendency to automatically mimic and synchronize expressions, vocalizations, postures, and movements with those of another person’s and, consequently, to converge emotionally” [11]. Emotional contagion is a well-known basic building-block of empathy [5] and implies that two (or more) subjects share the same affective state [12]. The perception of others’ action leads the observer to automatically reproduce the same action. Through this unconscious mirroring response, the observer can experience the same affective emotional state underpinning such action [13]. 

The expression and perception of emotions in non-human animals seem to be adaptive because these skills allow them to respond quickly and appropriately to unpredictable situations thus increasing their survival and fitness [14,15]. According to the perception–action coupling, the capacity to mirror others’ behaviors acquires an even more adaptive value in animals whose relationships are not inhibited by rank rules and that build and maintain their bonds through cooperation and social affiliation [16]. 

Beyond primates, cohesive and cooperative societies also characterize several social carnivores such as wolves (*Canis lupus lupus*) and dogs (*Canis lupus familiaris*) [17,18]. Compared to wolves, dogs have expanded towards humans their propensity to synchronize and affiliate [2]. Dogs develop strong affinitive bonds with owners, which represent their favorite social partners [19]. One of the aspects of this preferred affectional bond seems to be the empathically predisposition of dogs towards humans’ emotions [20]. The capacity of dogs to understand and experience humans’ emotions (i.e., empathy) [5,12] can be evidenced by their (1) preferential responses to the cry of humans compared to their humming or talking [21], (2) ability to discriminate emotions that are more positive for owners [22] and (3) high propensity to be infected by humans’ yawns [23,24,25]. The question arising from these evidence deals with the origin of the human–dog emotional sharing: is it a phenomenon shaped by artificial selection or evolutionarily rooted in the line of social carnivores? Trying to answer the question, we focus on rapid mimicry (RM) and yawn contagion (YC) in wolf and dog groups to investigate if the ability to emotionally engage with conspecifics can provide the basis for the development of inter-specific emotional sharing.

## 2. Social Alignment: Motor and Emotional Synchrony

Although rapid mimicry (RM) and yawn contagion (YC) are different behavioral phenomena, there is evidence that they rely on similar mechanisms. In this theoretical framework, the dyad composed by interacting subjects is considered as an “interactive unit” in which coordinating actions and possibly shared emotions create a shared identity [26,27]. Reaching a motor and emotional synchrony requires a social alignment, which is sustained by a feedback mechanism involving three different components. The first component is represented by the capability to perceive the presence of a non-alignment social system deriving by a non-congruent emotional and motor response with the partner. The second component involves the skill to adjust the non-congruent response to achieve a social alignment during dyadic interactions. This alignment stimulates brain areas associated with positive reward (the third component) that can be related to the sense of satisfaction occurring when an experience connectedness is reached [26]. In this view, a simple congruent motor response (e.g., RM, YC) operating in an alignment social system can produce a domino effect that, by fostering the creation of dyadic social bonds, produces complex and cooperative social networks.

### 2.1. Rapid Mimicry: The Key for Fair Play and Bonding Success

Play may represent one of the most fertile social contexts in which the alignment–reward system [26] is unveiled. Due to its autotelic nature, play is per se a rewarding activity for animals [28,29]. Motor synchronization allows animals to avoid any kind of misinterpretation and prolong the playful interaction thus maximizing rewarding [30]. Indeed, play induces a positive emotional state that can be shared with the playmate through specific forms of motor resonance defined rapid mimicry (RM) [31]. RM is an unconscious, congruent, and rapid response (less than 1 s) during which human and non-human animals automatically mimic others’ expressions and movements [13,32]. RM has been demonstrated not only in humans but also in several primate [33,34,35,36], rodent [27,37], and carnivore species [31,38,39].

Considering canids, RM has been found in dogs [31]. During intraspecific play, two playful meta-communicative signals can trigger fast and mimic responses: the play bow and the relaxed open mouth [31]. Generally, play signals are performed by playmates for communicating their positive "intentions" thus avoiding misunderstanding and escalation into overt aggression [40]. When performing a play bow, dogs crouch on their forelimbs, remain standing on their hind legs and wag their tails while sometimes barking; this behavioral pattern allows the individual to place its head below the playmate in a nonthreatening position [41]. The bow is a steady posture from which the subject can easily change its position and, for this reason, Pellis and Pellis [42] suggested that it can function as a strategic posture that permits the bower to launch an attack on the partner and/or better escape from the playful arena. As regards playful facial signals, the relaxed open mouth is considered the ritualized version of the play bite [43,44,45]. It can be performed by dogs at different gradients; indeed, the mouth can be opened just a little revealing only the upper parts of the lower jaw or in a wider way completely revealing the lower and upper jaws [46]. 

A recent study [47] examining social communication in dogs supports the findings provided by Palagi et al. [31]. Howse and coworkers [47] demonstrated that not only play bows but also pull–rear away patterns were reciprocated by playmates. Although the authors did not focus on the exact initiation and replication of these playful patterns, the data suggest that play bows were mimicked and that the pull–rear away pattern should be worth to be considered as a communicative signal due to its involvement in dog social mimicry. Interestingly, both play bows and pull–rear away patterns were not correlated with any other behavior received by the trigger thus suggesting that these specific signals might induce a mimicry response in dogs. This study opens new scenarios on play communication in dogs by highlighting the role of mimicry in unveiling the communicative nature of selective motor actions that have been often considered as simple playful patterns. 

After demonstrating that both play bow and relax-open mouth were rapidly mimicked by the players, Palagi and co-authors [31] showed that the length of the session, a reliable measure of play success also in dogs, was positively linked to the presence of the mimicry phenomena. Specifically, those playful sessions punctuated by RM lasted more compared to the sessions characterized by the mere presence of at least one of the two signals not followed by mimicry. According to the Bekoff’s hypothesis [48] about the importance of social play in the evolution of animal morality, it is possible that RM can be a building block at the basis of play fairness and trust between the subjects. At a short-term, sharing the playful mood through RM can create an emotional bridge that may foster cooperation and self-reward in the players. At a long-term, prolonged and fair playful interactions permit individuals to make “more training” for improving their communicative and social competence under safe circumstances [49]. Interestingly, Romero and colleagues [50] demonstrated a positive effect of oxytocin (a peptide hormone produced in the hypothalamus) in prolonging the play session. When the authors sprayed the dogs with oxytocin, the animals not only were more motivated in initiating play, but they also engaged in longer playful sessions. Even if very few studies are available on the relation between oxytocin and rapid mimicry in non-human animals, physiological studies in humans demonstrated that this hormone enhances inter-brain synchrony [51] and motor mimicry (e.g., pupil dilation and constriction) [52] during social interactions and coordination. What we know up to now is that RM prolongs the duration of the playful interactions between dogs [31] and that the administration of oxytocin has a similar effect on dog–human play [50]. The new challenge is to evaluate if the administration of oxytocin can increase motor mimicry (RM) in dogs both at intra- and inter-specific level that, in turn, favors the success of play by prolonging the session. 

A further important issue to explore would be whether the administration of oxytocin increases the mimicry response also when the interacting subjects are unfamiliar. Indeed, as it occurs in humans [9], the levels of RM during dog play followed an *empathic gradient* being greatest in response to friends, then acquaintances, and lastly strangers. As pointed out, play offers a positive and safe “environment” in which subjects can implement not only their physical skills, but also their emotional capacities [53]. The ability of perceiving and sharing others’ emotional states permits the players to anticipate partners’ intentions and modulate their actions consequently. In this light, RM can represent an affinitive signal that may function in strengthening the social bonds between interacting subjects [54]. Even if humans represent the “best friends” of dogs [19], during intra-specific play, especially play fighting, individuals need to be “emotionally linked” for better synchronizing their behaviors and emotions thus interacting successfully. 

**Figure 1:**
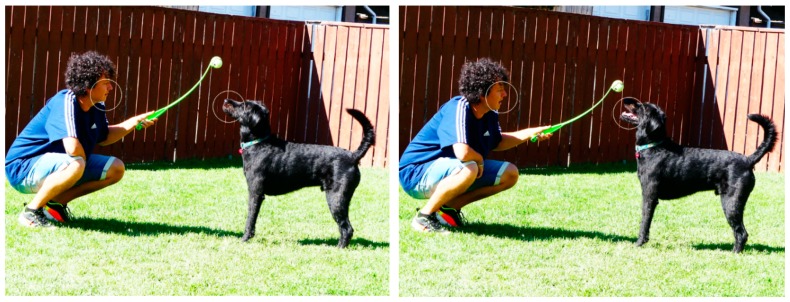
An example of facial mimicry between humans and dogs. Although it is difficult to know who mimics whom, the congruent response during play with object was extremely fast (two frames, 2-csec accuracy). Photo by Elisabetta Palagi.

Up to now, no quantitative data are available on dog–human facial mimicry during play, even though some anecdotic observations seem to suggest the presence of the phenomenon (Figure 1). 

However, other forms of mimicry have been investigated between dogs and their owners; for example, Sümegi et al. [55] found not only that emotional contagion for stress exists between dogs and their owners but also that it provokes changes in the dogs’ memory performance. Some findings also exist on the long-term stress synchronization [56] thus revealing a hormonal mimicry already reported for interactions between humans [13]. Moreover, mimicry can involve physiological and psychological factors which are strongly socially dependent. In a recent paper, Katayama and coworkers [57] demonstrated that the heart rate variability (HRV) of dogs and their owners, both tested under stress and control conditions, was positively correlated with the length of dog ownership. The authors suggested that the emotional contagion between humans and dogs may be linked to the amount of time the two subjects spend in sharing the same environment. 

Motor mimicry often implies an eye-to-eye contact between the interactans that can have consequences in creating an emotional bridge between partners. Nagasawa et al. [58] demonstrated that dog–owner mutual gazing strongly affected the oxytocin levels of both subjects by fostering a positive “chemical and affiliative mimicry” loop. Human-like communicative characteristics permit dogs to engage in complex and cooperative interactions with humans; the mutual eye contact represents a strong addressing signal for dogs and it can be a successful means of expressing human communicative intent [59].

Taking together, all these findings suggest that motor, physiological, psychological, and hormonal mimicry phenomena are intertwined factors that may depend more on social closeness than on phylogenetic relatedness. This shared interspecies communication, which is at the basis of social attachment, could be a promising starting point for expanding the investigation of motor resonance phenomena (e.g., rapid facial mimicry) between dogs and humans. Interestingly, mutual gazing or chemical/motor mimicry between hand-raised wolves and their care-takers were not confirmed [58]. Are wolves really not able to engage in motor resonance response and emotional sharing when interacting with others? 

Miklósi and Topál [60], pointed out the role of domestication process in causing behavioral changes in dogs. These changes affect the *Evolutionary Social Competence* that is the complex system of specific prosocial and coercive behavioral skills showed by interacting individuals of a given species. Probably, wolves with more human-like social competence gradually increased the closeness and affiliation with humans which, in turn, positively evaluated the presence of a companion animal with well-matched social and communicative skills. It has been suggested that during dog domestication the reduction in the environmental stress and the increase in inter-specific social interactions could alter the expression of genes via an epigenetic modulation that led to changes in social brain systems and behaviors [61,62]. 

To reach a clear picture of the evolution of rapid mimicry phenomena we need to understand if they have been shaped by the artificial selection operated by humans or if they are evolutionarily rooted in the line of social carnivores. The next challenge is to focus on the occurrence and social modulation of the rapid mimicry phenomena during intra-specific interactions in wolves. This will help understand the proximate causes of the evolutionary processes at the basis of the development of automatic mimicry and synchronization skills not only in dogs’ ancestors but also in dog–human communication exchange. 

### 2.2. Yawn Contagion in Dogs and Wolves: When the Partner Matters

Spontaneous yawning is a primitive stereotyped motor action pattern, that once triggered, is uncontainable and unstoppable [54]. It cannot be considered as a facial expression because it recruits facial, oral, laryngeal, pharyngeal, thoracic and abdominal muscles. Indeed, depending on the species, yawning may be also accompanied by eye closing, vocalizations, body stretching, pandiculation and even tongue protrusion [63]. Yawning is a widespread phenomenon in mammals and birds [64,65]. In humans, the new ultrasound technologies evidenced the presence of spontaneous yawning just starting from the fetal phase [66]. The occurrence of fetal yawning seems to contribute in assessing the correct development of brainstem and understanding the neural underpinnings of sleep and arousal systems [66]. Several hypotheses on the functions of spontaneous yawning in non-human animals have been formulated and these involved both physiological and social explanations. Indeed, yawning could act as a homeostatic restoring and brain cooling mechanism, an anxiety and drowsiness signal, and it has also a role in the social communicative system (e.g., threat or alertness yawn) [63].

Contagious yawning, a behavioral act involuntarily induced by viewing or listening other’s yawns [64], is considered as a different phenomenon which has been widely demonstrated in human and non-human primates [67,68,69,70,71]. In primates, yawn contagion can be considered as a proxy of emotional contagion and it can be associated with the level of social attachment between partners [5]. Even though in human and non-human primates the social modulation of contagious yawning is supported by ethological [68,69,70,71], neurological [72,73], and psychological evidence [74], contradictory results about proximate mechanisms of contagion emerged from the studies on domestic dogs. 

In 2008, Joly-Mascheroni and co-authors [23] evidenced that dogs responded to humans’ yawns in the 72% of experimental cases. This first evidence of contagious yawning between two different species led to hypothesize a possible association between the phenomenon and empathic involvement between partners. Later, two studies found neither evidence of yawn contagion in dogs nor its linkage with empathy [75,76]. In 2013, Madsen and Persson [77] demonstrated the occurrence of dog–human yawn contagion. However, the empathic interpretation of the phenomenon was not supported because the relationship quality shared by the interacting subjects (dog-owner vs dog-unfamiliar human) did not affect the level of contagion [77]. Silva and colleagues [25] provided evidence in favor of the empathic hypothesis of contagious yawning in dogs by using auditory instead of visual stimuli. During the experiment, dogs yawned more frequently when the auditory stimulus came from familiar (the owner) rather than unfamiliar humans. This first evidence of social modulation of yawn contagion was quickly challenged. In many species, including dogs, spontaneous yawning can emerge in response to environmental or social stressors [63,78,79,80]. In this light, it has been suggested that the finding obtained by Silva and colleagues [25] could be simply ascribed to a distress response by dogs. When dogs heard, but not saw, their owner, they experienced high anxiety levels that determined an increase in their yawning response.

In order to discriminate between the two possible proximate causes (empathy vs anxiety) of yawn contagion in dogs, Romero and co-workers [24] carried out an elegant study integrating ethological and physiological approaches. During the research, dogs observed familiar (the owner) and unfamiliar humans while yawning (experimental condition) or simply making mouth movements (control condition). Concomitantly, the heart rate and heart rate variability, two reliable physiological indicators of stress and well-being in animals [81], were monitored. The authors demonstrated that dogs yawned more frequently in experimental than in control condition and they were more infected by the yawns of familiar than unfamiliar humans. Intriguingly, the physiological parameters did not differ between experimental and control condition, thus suggesting that yawn contagion was not related to environmental or social stressors, but it was modulated by the affinitive attachment between dogs and their owners.

Recently, by investigating the possible relation between yawn contagion and the empathic gradient Kis and colleagues [82] provided contrasting results. The authors tested 33 dogs in both yawning and control condition by previously treating them with intranasal oxytocin or placebo. Contagious yawning did not occur at all and the number of yawns performed by dogs were not related to the degree of social closeness with owners but rather with the levels of anxiety measured by mouth licking. Moreover, the oxytocin pre-treatment significantly reduced the yawning events. Nevertheless, it is worth noting that intranasal administration of oxytocin can activate the endogenous hypothalamic oxytocin system, thus the still lack of information about the neural, physiological and behavioral consequences of this activation implies to interpret with caution the observed effects of exogenous oxytocin [83,84,85]. Accordingly, Romero and colleagues [50] found that the effects of exogenous oxytocin in dogs were fine-tuned by the individual variability in endogenous oxytocin: the dogs showing high basal levels of oxytocin were less responsive to the effects of intranasal oxytocin. In order to clarify the "social role" of oxytocin in dog–human emotional contagion, future studies should take into account the interaction between the applied hormone doses and endogenous secretion in modulating behavioral changes.

If the occurrence of contagious yawning and its proximate causes have been investigated in the dog–human relationship, no studies are available on the phenomenon of yawn contagion between dogs. Is the phenomenon of yawn contagion exclusively linked to dog–human interactions because of the domestication process or is this motor resonance phenomenon deeply rooted in social carnivores and simply transferred from conspecifics to human partners in the domestic dogs?

A study carried out by Romero and colleagues [86] on captive wolves may answer the question. The authors observed the animals in their environmental and social setting without altering their ordinary habits. The wolves yawned more frequently when they were exposed to conspecific yawning and the propensity to be infected was positively linked to the strength of social relationship shared between the trigger (the initial yawner) and the observer. These findings suggest that contagious yawning is an ancestral phenomenon firstly naturally selected for intra-specific social communication and group synchronization and later artificially selected for inter-specific communication. To complete this framework, it is therefore crucial exploring the occurrence of the phenomenon of yawn contagion and its potential roles in groups of dogs characterized by a certain level of variance in the distribution of relationship quality. 

## 3. Conclusions

Wolves, the ancestors of dogs, constitute cohesive family group whose members actively and cooperatively participate to the pack life creating a sort of “division of labor” system [87,88]. As humans, wolves engage in strong social and emotional bonds with conspecifics and these relations could be at the basis of the evolution of dog–human affective attachment [62]. The brain areas involved in reward, stress, imitation, and prosocial systems have been evolutionary conserved along with the developmental pathway of mammals [89,90,91]. This common neurological state could have permitted wolves and (ancient) humans to develop, during the progressive increase of their cohabitation, a shared representation of actions and emotions. Motor and emotional sharing probably promoted wolf–human social interactions and improved interspecific empathic predisposition [92] that is at the basis of the long history of dog–human affinitive relationship. 

In conclusion, to understand the role of the domestication process and artificial selection operated by humans in shaping the emotional contagion of dogs (rapid mimicry and yawn contagion), we should focus not on the possible presence and distribution of these phenomena between hand-reared wolves and humans but, instead, between the different wolf pack members [93]. Long-term studies should quantify (dyadic variance, seasonality, hormonal variations) and qualify (affiliation, agonistic support) the relationship quality shared by the subjects and verify whether the incidence of rapid mimicry and yawn contagion is socially modulated in wolves. A further important point is whether the occurrence and the distribution of rapid mimicry and yawn contagion covariate with some empathy-driven and prosocial behaviors such as consolation and helping. This could provide an indirect evidence of the potential empathic nature of these motor resonance phenomena.

As for dogs, we should focus on the different breeds. By selecting the most recent (less human-oriented) and ancient breeds (more human-oriented) as models we can evaluate to what extent the artificial selection has operated in shaping the mimicry phenomena. To reach the goal, observational studies on groups of dogs belonging to the same breed and experimental studies including dogs of different breeds and their owners are necessary.
